# Longitudinal auditory data of children with prelingual single-sided deafness managed with early cochlear implantation

**DOI:** 10.1038/s41598-022-13247-5

**Published:** 2022-06-07

**Authors:** Tine Arras, An Boudewyns, Freya Swinnen, Andrzej Zarowski, Birgit Philips, Christian Desloovere, Jan Wouters, Astrid van Wieringen

**Affiliations:** 1 Department of Neurosciences, Experimental Oto-rhino-laryngology, KU Leuven, O&N2, Herestraat 49, box 721, 3000 Leuven, Belgium; 2 Cochlear Technology Center, Schaliënhoevedreef 20i, 2800 Mechelen, Belgium; 3 Department of Otorhinolaryngology, Antwerp University Hospital, Drie Eikenstraat 655, 2650 Edegem, Belgium; 4 Faculty of Medicine and Translational Neurosciences, University of Antwerp, Universiteitsplein 1, 2610 Wilrijk, Belgium; 5 Department of Otorhinolaryngology, Ghent University Hospital, C. Heymanslaan 10, 9000 Ghent, Belgium; 6 European Institute for ORL-HNS, Sint-Augustinus Hospital Antwerp, Oosterveldlaan 24, 2610 Wilrijk, Belgium; 7 Department of Otorhinolaryngology, Head and Neck Surgery, University Hospitals Leuven, Herestraat 49, 3000 Leuven, Belgium

**Keywords:** Paediatric research, Paediatric research

## Abstract

Individuals with single-sided deafness (SSD) have no access to binaural hearing, which limits their ability to localize sounds and understand speech in noisy environments. In addition, children with prelingual SSD are at risk for neurocognitive and academic difficulties. Early cochlear implantation may lead to improved hearing outcomes by restoring bilateral hearing. However, its longitudinal impact on the development of children with SSD remains unclear. In the current study, a group of young children with prelingual SSD received a cochlear implant at an early age. From the age of four, the children’s spatial hearing skills could be assessed using a spatial speech perception in noise test and a sound localization test. The results are compared to those of two control groups: children with SSD without a cochlear implant and children with bilateral normal hearing. Overall, the implanted group exhibited improved speech perception in noise abilities and better sound localization skills, compared to their non-implanted peers. On average, the children wore their device approximately nine hours a day. Given the large contribution of maturation to the development of spatial hearing skills, further follow-up is important to understand the long-term benefit of a cochlear implant for children with prelingual SSD.

## Introduction

People with bilateral normal hearing (NH) use interaural differences in time (ITD) and level (ILD) to derive spatial characteristics of a sound. Binaural processing is crucial for accurate sound localization and contributes strongly to speech perception in noisy environments^1^. For listeners with single-sided deafness (SSD), i.e. individuals with normal hearing (NH) in one ear and profound hearing loss (> 90 dB HL) in the other ear, only monaural localization cues (e.g. spectral cues) are available^2^. As a result, they may experience degraded speech perception in noise, impaired sound localization abilities and increased listening effort^3–6^, all of which can have a lasting impact on psychological wellbeing^7^. In addition, children with prelingual SSD are at risk for academic underachievement, speech-language delay, socio-emotional difficulties and problems with complex cognitive skills^8–10^. These limitations may not resolve with age without intervention^11^. Untreated SSD can also result in the aural preference syndrome, in which cortical processing is altered in favor of the normal hearing ear^12,13^. Early treatment, during a period of high neuroplasticity, is expected to prevent this asymmetry or restore normal processing^14^.

Traditional treatment options for SSD include contralateral routing of signal (CROS) hearing aids and bone conduction devices (BCDs). In both cases, sound awareness from the deaf side is restored by providing additional auditory input to the contralateral ear. In contrast, cochlear implants (CIs) activate auditory nerve fibers in the ipsilateral cochlea to restore sound perception. As such, this is the only treatment option that enables bilateral auditory stimulation, which is expected to facilitate better auditory processing as well as improved cognitive and spoken language performance^15–18^. Although a CI can restore bilateral sound perception in listeners with SSD, binaural hearing may be difficult to achieve for multiple reasons. Previous research in adults with SSD and a CI indicated a frequency-dependent timing mismatch for acoustic compared to electric stimulation of the auditory nerve, with the CI activating the cochlear nerve several milliseconds earlier compared to typical acoustical processing^19,20^. Given that interaural time differences (ITDs) are typically below one millisecond^1^, this delay is expected to disrupt ITD perception, leaving only interaural level differences (ILDs) as a cue for localization. However, it is unclear to what extent ILD cues are preserved, given the imbalance between the wide dynamic range of a normal hearing ear compared to the limited dynamic range that the CI offers. In addition, interaural place-of-stimulation mismatch may prevent accurate comparison of frequency information between both ears, causing further deterioration of the binaural cues^21,22^.

As a result, it is expected that providing listeners with SSD with a CI will improve their speech perception in noise, however the size of this improvement depends on the spatial relationship between the speech and noise sources. In terms of sound localization, prolonged experience with the combined electro-acoustic stimulation as well as dedicated training could lead to improved performance^23,24^. For children, early intervention, during a period of high neuroplasticity, may enable optimal outcomes^13,25^.

In a recent randomized controlled trial comparing CROS, BCD and CI in adults with acquired SSD, cochlear implantation resulted in the largest improvements in spatial speech perception in noise, sound localization, tinnitus relief, and hearing-related quality of life^26^. Similar outcomes have been reported in numerous studies regarding cochlear implantation for SSD in adults^27–33^. Previous research in children with SSD suggests that the duration of deafness affects the auditory outcomes after cochlear implantation, with less favorable results after a longer period of untreated SSD^34,35^. In general, children with SSD demonstrate improved speech perception in noise and sound localization after implantation, similar to adults^36–39^. Additionally, most children use their CI regularly and understand speech with the CI alone^40–43^.

Unfortunately, while these studies reported significant benefits after cochlear implantation, it remains difficult to generalize their results. Most studies had some limitations, for example heterogeneous participant groups (including variable duration and cause of deafness), few assessments during a short follow-up period, or lack of control groups. To address some of these limitations, a longitudinal multicenter study was set up in Belgium. In this study, a group of fifteen children with prelingual SSD received a CI before the age of 2.5 years. The age restriction allowed for limited duration of deafness and initial CI activation during a period of high cortical plasticity, both of which were expected to positively impact outcomes. The study also included two control groups: 17 children with prelingual SSD without a CI and 30 children with bilateral NH. Adding these control groups was expected to help disentangle the effects of maturation versus intervention, and to compensate for potential recruitment bias. An ongoing longitudinal study with regular auditory and neurocognitive assessments is investigating the developmental patterns in each group.

Previously, we have shown that early cochlear implantation supports normal language development, while untreated SSD may lead to delays in language development, especially with regard to grammar^44,45^. In this study, we investigate the children’s spatial speech perception in noise and sound localization abilities. These skills can be reliably assessed from the age of 4 years^46^. Currently, the majority of the participating children is old enough to participate in these auditory-behavioral tests. Both speech perception in noise and sound localization abilities rely heavily on binaural processing, in particular on ITD and ILD perception. While these binaural cues are likely distorted due to the mismatch between the acoustic and CI stimulation, young children may learn to use them if the CI is activated within the period of high cortical plasticity. Additionally, the mere provision of bilateral input and the associated alleviation of the head shadow effect may already support better spatial hearing. As such, we hypothesized that early cochlear implantation would lead to improved speech perception in noise and better sound localization in young children with prelingual SSD. In addition to these auditory tests, we investigated daily device use in the group of children with a CI. Consistent device use likely contributes to successful processing of the CI input, and therefore it may promote improved auditory outcomes^47,48^.

## Methods

The study was designed and conducted according to the Declaration of Helsinki. The study protocol was reviewed and approved by the medical-ethical committee of the University Hospitals Leuven (registration number: B322201523727) and at all participating centers. Written informed consent was obtained from the parents of all participants.

### Participants

Based on the required minimum age of 4 years to participate in the tests, the current report includes only 21 of the 32 children with SSD in our sample. The children were recruited from four academic hospitals in Belgium between 2015 and 2018. At the time of inclusion, click-evoked auditory brain stem response (ABR) audiometry had confirmed that all children presented normal hearing thresholds in one ear (ABR ≤ 35 dB nHL) and severe-to-profound hearing loss in the contralateral ear (ABR ≥ 80 dB nHL). In all but one case, the hearing loss was present at birth. Children were considered for cochlear implantation if they had an intact auditory nerve, confirmed by magnetic resonance imaging of the posterior fossa, and were younger than 2.5 years. As such, 12 children in the current study received a CI, with a mean age at implantation of 13.6 ± 4.6 months. One child experienced a progressive hearing loss in the better ear, approximately one year after implantation, for which it was fitted with a hearing aid. The noCI children were not implanted due to cochlear nerve deficiency (CND, n = 5), their age at the time of inclusion (too old for CI, n = 2), or lack of parental consent (n = 2). Additional child characteristics for both groups are presented in Table 1. The control group consists of 26 normal hearing (NH) children (15 male, 11 female). All NH children were born between 2013 and 2017.

**Table 1:** Child characteristics for the SSD groups.

Characteristic	SSD + CI group (n = 12)	SSD-noCI group (n = 9)	Total (n = 21)
**Sex**			
Female	5 (42%)	3 (33%)	8 (38%)
**Side of hearing loss**			
Left	7 (58%)	5 (56%)	12 (57%)
**Cause of deafness**			
Congenital cytomegalovirus infection	8 (67%)	2 (22%)	10 (48%)
Cochlear nerve deficiency	0 (0%)	5 (56%)	5 (24%)
Cochlear malformation (incomplete partition type II)	1 (8%)	0 (0%)	1 (5%)
Petrous bone fracture (at age 10 months)	1 (8%)	0 (0%)	1 (5%)
Unknown	2 (17%)	2 (22%)	4 (19%)

All children in the SSD + CI group received a Cochlear Nucleus implant (CI522, n = 6; CI532, n = 4; CI512, n = 1; CI422, n = 1) and sound processor (N6, n = 6; N7, n = 3; Kanso, n = 3). So far, two children upgraded from an N6 to an N7 sound processor at approximately 5 years after initial activation. All devices were set to monopolar stimulation modes with two extracochlear return electrodes (“MP1 + 2”), along with ACE (Advanced Combinational Encoder) speech coding strategies with eight (n = 9) or ten (n = 3) maxima. In the MAPs (i.e., the individual processor settings that map acoustic sounds to electrical stimulation) of two children, the two or three most basal electrodes were deactivated. For all children but one, the automatic scene classifier system was activated for all listening programs. For the remaining child, standard directionality was selected instead. The children received no specific rehabilitation within the framework of the study.

### Data collection

The ENT teams from the different hospitals provided clinical and audiometric data for the 21 children with SSD. Data logs containing the average daily CI use were extracted from the Custom Sound Pro fitting software (Cochlear Ltd.). We defined daily CI use as the average time-on-air value across the three most recent data logs per child.

All 47 children participated in two auditory perception tests: spatial speech perception in noise and sound localization. Children were typically tested once a year, with both tests planned consecutively on the same day. The children with a CI were tested twice within one month, to assess both their aided (session 1) and unaided (session 2) performance on the tests. During the aided test session, the sound processor was set to the default program, to match the children’s daily listening experience. Prior to auditory testing, normal hearing thresholds were confirmed by the children’s clinical records or based on pure tone audiometry.

### Spatial speech perception in noise

The LittleLINT speech material was used to assess spatial speech perception in noise^18^. It consisted of numbers 1–10, which had been extracted from the Leuven Intelligibility Number Test (LINT) speech material^49^. During the task, continuous speech-weighted noise was presented at a fixed level of 55 dB sound pressure level (SPL) in free field. The speech level was adapted in steps of 2 dB to determine the speech reception threshold (SRT): the speech-to-noise ratio (SNR) at which the child could understand approximately 50% of the speech. The SRT was calculated by averaging the SNR of the last five trials together with the SNR for the virtual next trial (based on the SNR of the last trial and the child’s response). The children were asked to repeat the number and received positive reinforcement independent of their answer.

**Figure 1:**
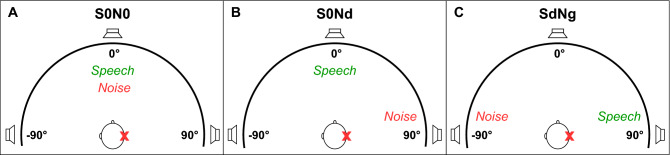
Schematic representation of the spatial conditions for the speech in noise test. Stimuli were presented at 0° azimuth (‘0’), at 90° azimuth on the NH (good) side (‘g’), or at 90° azimuth on the deaf side (‘d’). Participants were seated in the middle between the loudspeakers (1 m distance), with their ears at the same level as the loudspeakers. They were instructed to face the loudspeaker at 0° azimuth.

The speech perception test included three spatial conditions (see Fig. 1): co-located speech and noise at 0° azimuth (S0N0); speech at 0° azimuth and noise at 90° on the deaf side (S0Nd); and speech (90°) at the deaf side and noise (90°) at the NH (‘good’) side (SdNg). For children with bilateral NH, noise was presented on the right side in the second condition (S0Nd) and on the left for the third condition (SdNg, with speech on the right side). Children were facing the loudspeaker at 0° azimuth during the entire test. For each spatial condition, at least two lists were completed. A list consisted of ten words, with each number occurring once in random order. If the number of reversals was too low (< 4), the standard deviation (SD) of the SRT was too high (> 2 dB), or the difference between the SRTs of both lists was too high (≥ 5 dB), an additional list was presented in the same condition. Afterwards, the average SRT was calculated for each spatial condition. For children with SSD and a CI, both aided and unaided performance was quantified and compared across conditions.

### Sound localization

**Figure 2:**
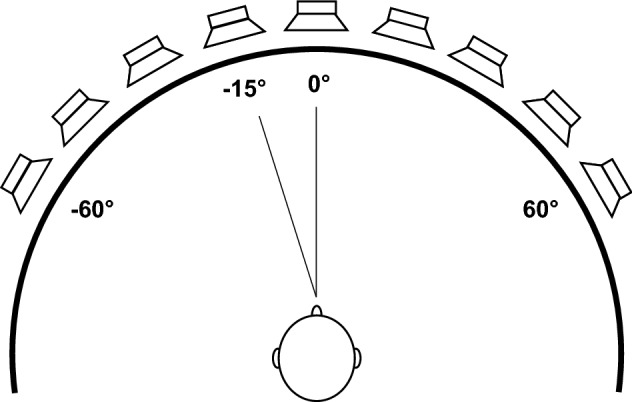
Test set-up for the sound localization test. The loudspeaker array consisted of nine loudspeakers ranging from −60° to + 60° azimuth, spaced 15° apart. Participants were seated in the middle of the array, with their ears at the same level as the loudspeakers. They were instructed to face the loudspeaker at 0° azimuth.

The sound localization test was based on the protocol described by Van Deun et al.^46^. The child was seated in the middle of a loudspeaker array with nine speakers, ranging from −60° to 60° with a 15° interval (see Fig. 2), facing the loudspeaker at 0° azimuth. A one-second telephone sound was presented from one speaker at a time. The stimulus presentation level was roved between 59 and 65 dB SPL (i.e., sounds were presented at 59, 62 or 65 dB SPL) to avoid the use of sound level as a cue for localization. The localization performance was quantified for each child using the mean absolute error (MAE, in degrees). This measure is more robust to large mistakes compared to the commonly used root mean square (RMS) error and is therefore more appropriate when testing children^46^. To calculate the MAE, the absolute difference between the stimulus and response angle (in degrees) was calculated for each trial, and subsequently averaged across all trials.

Given the young age of the participants, the speakers were labeled with pictures of Smurfs instead of numbers, to facilitate speaker selection. A researcher standing in front of the child (behind the loudspeaker at 0° azimuth) provided a visual cue before each trial (i.e., pressing a button on a toy telephone). The task began with a series of training sessions, with two, three or five loudspeakers, during which the children received no feedback on their performance. The two-loudspeaker training session was omitted for children with bilateral NH or those who had previous experience with the task. The training sessions were followed immediately by the test session with nine speakers. The test had a fixed number of three repetitions for each speaker, in a randomized order. The children received positive reinforcement independent of their performance.

### Statistical analysis

We used R version 4.0.3^50^ to analyze the data and to create the graphs. For all statistical tests, the significance level α was set to 0.05. Normal distribution of the results was assessed using Shapiro–Wilk tests. Both outcome measures were subsequently analyzed using linear mixed models (LMMs). Such models can take into account the clustering effect due to repeated testing of the same subjects by adding the subject’s identity as a random effect^51^. To analyze the data, four models were created: one for each spatial condition of the speech perception in noise test and one for the sound localization test. Each model included group as predictor, age as a covariate, and subject as a random effect: *result* ~ *group* + *age* + *I|ID.* The significance of each fixed effect was calculated using type II Wald chi-square tests. Post-hoc comparisons were performed using Tukey pairwise tests with Bonferroni correction, based on the estimated marginal means that were extracted from the corresponding linear mixed models.

## Results

Data for both spatial hearing tests (speech perception in noise and sound localization) were collected from 47 children, across three groups: 12 SSD + CI (median age 4.7 years, range 3.9 to 7.7 years), 9 SSD-noCI (median age 4.8 years, range 3.9 to 7.0 years), and 26 NH (median age 5.3 years, range 3.9 to 8.1 years). Most SSD + CI children had approximately 3 years of experience with their CI at the time of their first assessment (median time 3.1 years). Only the child with acquired SSD had less than 2.5 years of experience with the device when first tested (1.9 years). For each group, the median number of assessments was two per child (range 1 to 6). No outliers were excluded from the analyses. Average results per group are presented in the supplementary table.

### Speech perception in noise

**Figure 3:**
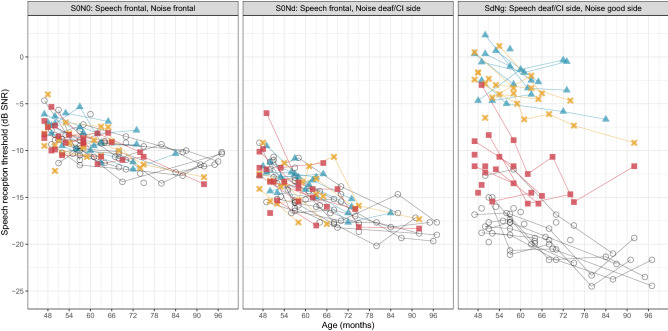
Individual averaged speech reception thresholds (in dB SNR) for the three spatial conditions as a function of age. SSD + CI (aided) = red squares; SSD + CI (unaided) = yellow crosses; SSD-noCI = blue triangles; NH = grey circles.

The purpose of this test was to investigate the possible benefit of the CI when speech and noise were presented at different locations. We expected that the CI would improve the SSD + CI children’s ability to perceive speech in noise, but that the improvement would depend on the spatial condition. Figure 3 shows the results of the speech in noise test for each spatial condition as a function of age. Each point represents the average SRT (in dB SNR) of an individual child at a certain test session. An alternative graph that compares group estimated means across conditions is included as supplementary figure A.

In the first spatial condition (S0N0), with speech and noise co-located at 0° azimuth, performance was similar across groups (*X*^2^(3) = 5.79,* p* = 0.12). Averaged over all participants, SRTs improved by 1.0 dB per year of age (*X*^*2*^(1) = 65.28,* p* < 0.001). The mean SRT for the NH group was −10.0 (± 1.8) dB. In the second spatial condition (S0Nd), with speech at 0° and noise presented to the deaf ear or CI, a similar pattern was observed: no effect of group (*X*^*2*^(3) = 4.62,* p* = 0.20), however a significant effect of age. On average across groups, SRTs improved by 1.7 dB per year (*X*^*2*^(1) = 130.23,* p* < 0.001). The mean SRT for the NH group was −15.5 (± 2.5) dB. Additionally, in the SSD + CI group, performance was similar between the unaided and aided condition. The mean difference between the aided and unaided SRT, based on pairwise comparisons within each child, was −0.1 dB (with lower SRTs in the aided condition), which was not significant (*t*(21) = −0.24,* p* = 0.81). This finding is illustrated in the middle panel of Fig. 3, as the aided (red boxes) and unaided (yellow crosses) results are in the same range.

In the third spatial condition (SdNg), with speech presented to the deaf/CI side and noise to the NH side, a similar improvement with age (1.6 dB per year, *X*^*2*^(1) = 79.53, *p* < 0.001) was observed. The mean SRT for the NH group was −19.2 (± 2.8) dB. Contrary to the previous conditions, SRTs were significantly different across groups (*X*^*2*^(3) = 1062.07, *p* < 0.001). Bonferroni-corrected pairwise comparisons confirmed that the children with bilateral NH had lower SRTs compared to all other groups (p < 0.001 for SSD + CI aided (−5.7 dB, *t*(53.1) = −9.60), SSD + CI unaided (−14.2 dB, *t*(55.9) = −23.96) and SSD-noCI (−16.2 dB, *t*(45.1) = −27.23)). For the SSD + CI children, aided performance was better than unaided performance (−8.5 dB, *t*(84.1) = −14.47,* p* < 0.001). With the CI, they outperformed their non-implanted peers (−10.5 dB, *t*(47.8) = −15.26,* p* < 0.001). Even in the unaided condition, their SRTs were slightly lower than those of the non-implanted group (−1.9 dB, *t*(49.9) = −2.82,* p* = 0.04). This difference might be explained by test familiarity, as the order of testing was always the same for the SSD + CI children (first the aided assessment, then the unaided assessment within one month). While the age-corrected difference between the NH group and the aided SSD + CI group was 5.7 dB (*t*(53.1) = −9.60, *p* < 0.001), visual inspection of the data showed that the highest (poorest) SRTs in the NH group were similar to those of the best SRTs in the aided SSD + CI group.

### Sound localization

**Figure 4:**
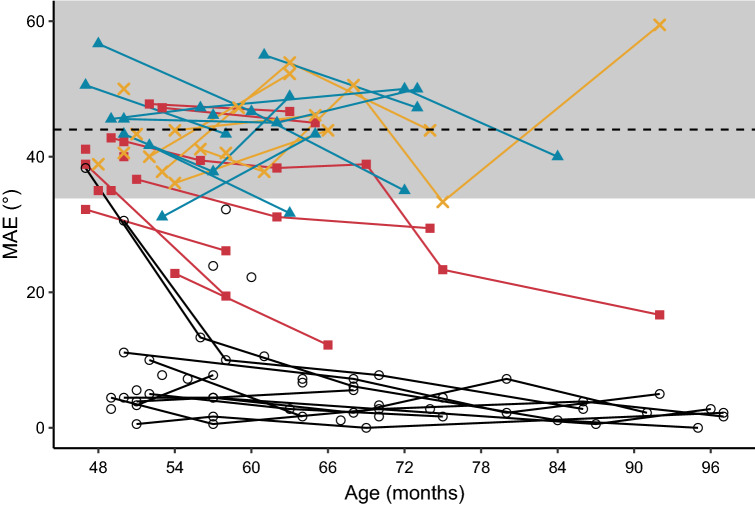
Individual mean absolute errors (in degrees) for the nine-loudspeaker condition as a function of age. SSD + CI (aided) = red squares; SSD + CI (unaided) = yellow crosses; SSD-noCI = blue triangles; NH = grey circles. The dotted line at 44° degrees corresponds to chance level performance; scores below the grey area are significantly better than chance. Red lines connect the better-than-chance scores of the SSD + CI children, to show developmental trajectories.

The aim of this experiment was to quantify the children’s sound localization abilities on the horizontal plane, for a nine-loudspeaker setup ranging from −60° to 60° azimuth. We expected that the CI would improve the children’s ability to localize sounds, i.e. that performance with the CI would be better than chance. The results of the sound localization test as a function of age are shown in Fig. 4. Additionally, a comparison of the group estimated means is presented in supplementary Fig. B.

Overall, MAEs improved with age (*X*^*2*^(1) = 13.21, *p* < 0.001), on average with 2.6° per year. The average MAE for the NH group was 6.7 (± 8.1) degrees. There was also a significant difference between groups (*X*^*2*^(3) = 412.02, *p* < 0.001) and an interaction effect between group and age (*X*^*2*^(3) = 15.86, *p* = 0.001). Post-hoc pairwise comparisons revealed significantly worse scores for all SSD groups when compared to the normal hearing controls (*p* < 0.001 for SSD + CI aided (24.9°, *t*(2.27) = 11.00), SSD + CI unaided (36.8°, *t*(53.8) = 16.13), and SSD-noCI (36.6°, *t*(43.4) = 16.33)). At group level, the SSD + CI children outperformed their non-implanted peers in the aided condition (−11.7°, *t*(47.4) = 4.34,* p* < 0.001). In the unaided condition, their performance was similar to that of their non-implanted peers (0.2°, *t*(49.2) = 0.08,* p* = 1), and much worse compared to the aided condition (11.9°, *t*(75.8) = 5.62,* p* < 0.001).

As shown in Fig. 4, five of the twelve SSD + CI children (aided condition) had scores below the cut-off for chance level performance, meaning that these children were able to localize sounds, albeit with relatively large errors. Three of these five children were initially unable to localize sounds, but gradually acquired this skill throughout their development. This is especially encouraging, as other children in the SSD + CI group may still be learning this skill.

Across all measurements, two SSD-noCI children (blue triangles) also performed better than chance. These children presumably used monaural loudness cues to estimate the location of the sound despite the intensity roving. Analysis of the data showed that louder sounds were localized to the side of the good ear, while softer sounds were localized to the side of the deaf ear. In addition, they may have relied on monaural spectral cues to estimate the location of the sounds. At group level, both the SSD-noCI group and the unaided SSD + CI group performed around chance level (44°).

Finally, a number of children with bilateral NH made relatively large errors at their first assessment (between the age of 4 and 5 years). This variability decreased strongly after the age of 5 years, and the children who initially made large errors also improved with age, to MAEs below 10°. This trend confirms that the proposed age limitation of the test of 4 years is the absolute minimum, and results may only be reliable from 5 years of age onwards.

### Daily device use

Data logging revealed that the SSD + CI children wore their speech processor on average 8.9 ± 2.7 h per day, with individual averages ranging between 2.9 and 12.2 h per day (see Table 2). At the time of the most recent read-out, the children had on average 3.9 years of experience with their device. Based on these records, 8/12 children are ‘regular’ users (≥ 8 h/day) and 4/12 are ‘limited’ users (< 8 h/day). The children’s device use did not correlate with their SRT or MAE scores.

**Table 2:** Average daily implant use at the three most recent data log readouts.

Child	Daily use (hours)	CI experience at last data log (years)	CI experience at first test (years)	Implant	Speech processor	Remarks
CI-01	2.9	5.4	1.9	CI422	Nucleus 6	Limited family support, recently became non-user
CI-02	12.2	5.7	3.5	CI522	Nucleus 6	
CI-03	10.6	3.9	3.2	CI522	Nucleus 6	
CI-04	6.4	4.3	3.3	CI522	Nucleus 6	
CI-05	9.9	4.5	3.1	CI522	Nucleus 6	
CI-06	10.1	4.0	2.7	CI532	Nucleus 7	Hearing aid in better ear (worn during all tests)
CI-07	7.1	4.0	2.9	CI522	Nucleus 6	
CI-08	10.2	3.5	3.1	CI532	Kanso	
CI-09	6.0	2.0	2.9	CI522	Nucleus 7	No recent data logs were available due to technical issues
CI-10	11.7	3.6	3.1	CI532	Kanso	
CI-11	9.3	3.3	3.2	CI512	Nucleus 7	
CI-12	10.1	3.2	3.0	CI532	Kanso	

The CI experience values reflect the time since the initial activation of the cochlear implant.

## Discussion

This study confirms that early cochlear implantation in young children with SSD significantly improves their speech understanding in noise and sound localization. As such, our data suggest that a CI may be beneficial to young children with SSD. For speech perception, a strong benefit was found in the aided compared to the unaided condition when speech was presented to the deaf ear. As a group, the children with a CI outperformed their non-implanted peers with an average advantage in SRT of 10.5 dB, which is a highly clinically relevant difference. Importantly, the CI did not hamper performance when noise was presented to the CI side. In terms of sound localization, five out of twelve children with SSD and a CI were able to localize sounds. At group level, this resulted in an average improvement of 11.7° compared to the non-implanted children. While these five children still made relatively large errors, most of them are still very young, and their localization abilities may improve with age. Indeed, even in the group of children with NH, some (of the younger) children are unable to perform the localization task with sufficient precision at the first assessment. Overall, these results suggest that providing bilateral hearing using a CI can have significant benefits for children with SSD. They are, however, insufficient to draw any definite conclusions about the restoration of binaural hearing in this population. Other factors, like head shadow alleviation or targeted noise suppression by the sound processor, could have contributed to the observed improvements in spatial hearing outcomes.

### Spatial hearing outcomes

Accurate sound localization requires accurate perception and processing of ITD and ILD cues^1,52^. As children with SSD cannot access such binaural cues, they are often unable to perceive the direction of sounds. While a CI in the deaf ear can restore bilateral sound perception, the various mismatches between the acoustical and electrical stimulation of the auditory nerve may still disrupt binaural processing. This might explain why some children with SSD and a CI are unable to localize sounds, despite having access to bilateral auditory information. Limiting the timing and frequency mismatch and improving the loudness balance between both ‘ears’ might promote better preservation of ILD cues, enabling better sound localization. Indeed, providing bimodal listeners with stronger ILD cues can improve their localization performance^53^. In addition, early cochlear implantation, during a period of high neuroplasticity, may allow the brain to make optimal use of the available cues. Furthermore, explicit training might improve the use of ILD cues for localization. To test this hypothesis, we are currently developing a binaural training game to assess and train the ILD processing of the children with SSD and a CI in our sample.

Children with SSD perceive the auditory world using one ear instead of two. While at first glance most of these children experience few difficulties during their development, their unilateral auditory impairment can significantly hamper their ability to learn and thrive. Being able to understand speech in the presence of noise can be particularly challenging for young children with SSD^3^, yet it is extremely important for their neurocognitive development. As young children typically spend considerable time in noisy environments^54,55^ they need sufficient speech-in-noise perception abilities to support their formal and informal learning. As such, providing these children with a CI may not only affect auditory outcomes, but it could aid their neurocognitive development as well. Indeed, we demonstrated that young children with SSD who received a CI show stronger grammar skills compared to their non-implanted peers^45^. This improvement is likely due to enhanced speech perception with the CI, which may especially affect the perception of short phonemes that carry grammatical cues (e.g. ‘sock’ vs ‘socks’, ‘work’ vs ‘worked’).

Device use is both an important factor for CI success and a relevant proxy for device acceptance. Children who wear their device regularly will learn to make optimal use of the available cues, and children who experience a benefit from wearing the device are more likely to wear it often. Data logs indicated that most children in our sample wore their implant on a regular basis, with an average of 8.9 ± 2.7 h per day. These numbers are slightly lower compared to the average of 9.8 h reported by Thomas et al.^38^, whose study included mainly older children with SSD and a CI (15 out of 21 participants were 4 years or older at implantation). In contrast, other studies including young children reported fewer hours of daily device use (6.5 to 7.4 h per day)^42,56,57^. Overall, daily device use is expected to increase with age^55,58^. Additionally, it can be influenced by multiple factors, including age at implantation, benefit from using the CI, type of education and maternal education level^59–61^. Recently, one of the children in our sample became a non-user, due to a lack of perceived benefit, despite measurable improvements on both spatial hearing tasks. This child had been a limited user since implantation (all-time average device use of 3.0 h per day), likely due to insufficient family support. Currently, none of the other children are non-users, suggesting that they derive at least some subjective benefit from wearing the CI. In addition, some parents reported that their child actively requests the CI when not wearing it.

Overall, the results reported here are in line with previous research. Earlier studies have shown that cochlear implantation can lead to improved spatial hearing outcomes^37,39^. Research suggests that superior outcomes are often found in children who received their CI early in life, compared to those who experienced a longer period of single-sided auditory deprivation^34,35^. To the best of our knowledge, so far only three studies investigating spatial hearing outcomes included only children with prelingual SSD^38,41,62^. These studies reported overall improved auditory-behavioral results and SSQ scores, but individual results varied widely across participants. The children’s age at implantation may explain some of this variation, as those ages ranged between 1.8–13.8 years^41^, 1.4–6.8 years^62^, and 0.8–11.3 years^38^, respectively. As such, some of these children far exceeded the recently proposed limit of 3–4 years of deafness for children with congenital SSD^34,35^. In contrast, participants in the present study all received their implant before the age of 2.5 years, which is well within the four-year limit. Indeed, all children in the current sample showed improved speech perception in noise.

### Strengths and limitations

The study design was chosen to address some limitations of previous research. Firstly, while most studies demonstrated clear benefits of cochlear implantation for children with SSD, many of them reported that one or more children in their sample experienced little or no benefit from the CI, without a clear explanation for this lack of improvement. Such outcome variability may be (partially) due to heterogeneity of the participant groups, for example with regard to onset and duration of deafness, cause of hearing loss, or age at implantation. To reduce this variability, the current study only included children with prelingual-onset SSD. They all received their implant at a young age (13.6 ± 4.6 months), which is within a developmental period of high neuroplasticity and was expected to optimize outcomes. Secondly, most studies were limited to one or a few consecutive assessments, which only provided a snapshot of the children’s development after cochlear implantation. The current longitudinal design was expected to provide a long-term view on the auditory benefit of a CI for these children. Additionally, the overarching study expanded the outcome measures to other developmental areas, including language and cognition, next to the standard auditory assessments. Finally, to the best of our knowledge, none of the previous studies included a control group of children with SSD without a CI. Unfortunately, comparing pre- and post-intervention assessments in the same group of children makes it difficult to disentangle the effects of maturation and intervention. Therefore, the current study included two control groups: children with SSD without a CI and children with bilateral NH.

The main limitation of the current study is the limited sample size. Given the strict selection criteria and the relatively low number of yearly cases of SSD in Belgium, the recruitment period for the current sample of 32 children with SSD spanned nearly 5 years. However, a power analysis confirmed that 15 children per group would suffice given the longitudinal study design.

## Conclusions

Despite the presence of a dominant normal hearing ear, providing a CI to children with prelingual SSD can have a significant positive impact on the children’s auditory development. The restoration of bilateral auditory input through the CI lead to improved speech perception in noise performance in our group of young children with prelingual SSD. Additionally, some children were able to localize sounds with reasonable accuracy. Improved spatial hearing, in turn, may support the development of other skills like grammar development^45^. As such, early cochlear implantation may offer many benefits to children with SSD and it should be considered as standard of care.

## Author contributions 

T.A.: Conceptualization, Data curation, Formal analysis, Funding acquisition, Investigation, Methodology, Project administration, Visualization, Writing—original draft. A.B.: Resources, Writing—review & editing. F.S.: Resources, Writing—review & editing. A.Z.: Resources, Writing—review & editing. B.P.: Supervision, Writing—review & editing. C.D.: Conceptualization, Resources, Writing—review & editing. J.W.: Conceptualization, Methodology, Writing—review & editing. A.v.W.: Conceptualization, Funding acquisition, Methodology, Project administration, Supervision, Writing—original draft, review & editing.

## Supplementary Information

Supplementary Information.

## Data Availability

The datasets generated during the current study are not publicly available due to privacy/ethical restrictions (as they contain information that could compromise the privacy of the participants). Anonymized data are available upon request from the corresponding author.
