# Supplementary material for: Longitudinal auditory data of children with prelingual single-sided deafness managed with early cochlear implantation
Source: Sci Rep. 2022 Jun 7;12:9376. doi: 10.1038/s41598-022-13247-5 (PMC9174487; doi:10.1038/s41598-022-13247-5)

*Supplementary table and figures for the manuscript “Longitudinal auditory data of children with prelingual single-sided deafness managed with early cochlear implantation”, by Tine Arras, An Boudewyns, Freya Swinnen, Andrzej Zarowski, Birgit Philips, Christian Desloovere, Jan Wouters, and Astrid van Wieringen.*

**Supplementary tables and figures**

Table: mean results for each group and outcome measure.

Figure A: individual SRTs taken together for all measurements.

Figure B: individual MAEs taken together for all measurements.

**Supplementary table**

Mean results for each group and outcome measure. SRTs are reported in dB SNR, MAEs in degrees.

Note that these values are not corrected for age, as opposed to the marginal estimated means reported in the pairwise comparisons in the main text.

| **Group** | **SRT S0N0** | **SRT S0Nd** | **SRT SdNg** | **MAE** |
| --- | --- | --- | --- | --- |
| NH | -10.0 (± 1.8) | -15.5 (± 2.5) | -19.2 (± 2.8) | 6.7 (± 8.1) |
| SSD-noCI | -8.4 (± 1.8) | -13.5 (± 2.0) | -2.1 (± 2.4) | 44.6 (± 6.6) |
| SSD+CI aided | -9.2 (± 1.7) | -13.8 (± 2.9) | -12.2 (± 3.0) | 34.5 (± 10.0) |
| SSD+CI unaided | -9.3 (± 2.0) | -14.1 (± 2.3) | -3.8 (± 2.4) | 44.0 (± 6.6) |

**Supplementary figure A**

Individual SRTs taken together for all measurements, for children from NH (grey circles), SSD-noCI (blue triangles), SSD+CI aided (red squares) and SSD+CI unaided (yellow crosses) groups. Estimated mean scores per condition (black dots) and corresponding confidence intervals (black bars) were added on top, based on models which analyzed within-group differences across conditions (*SRT_group_ ~ condition + age + I|ID*).

Within each group, all between-condition comparisons were highly significant (p < 0.001), except for the SSD+CI children in the aided condition (S0Nd-SdNg: *p* = 0.020). The graph does not show the effect of age, which was significant for all groups in all conditions.


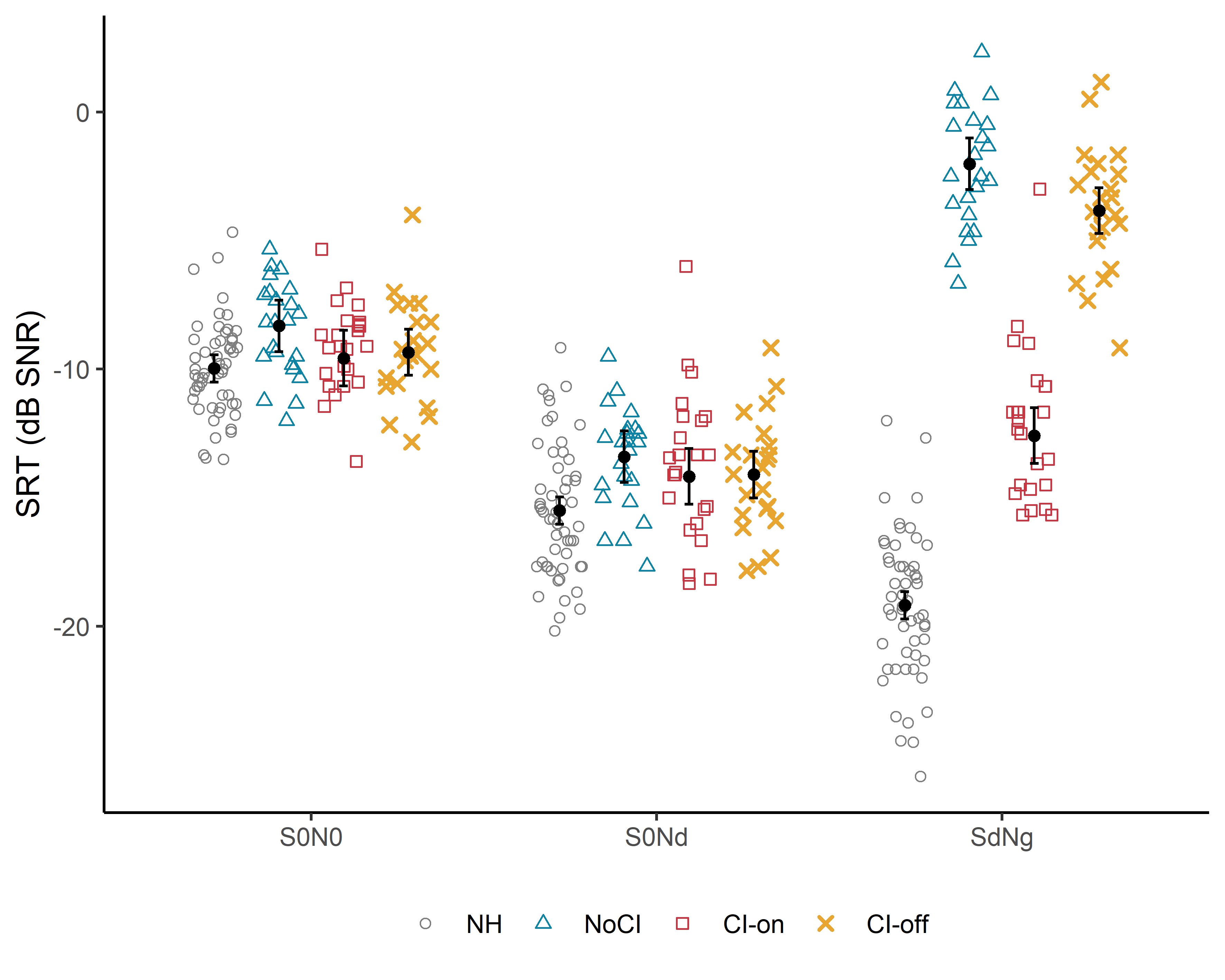


**Supplementary figure B**

Individual MAEs taken together for all measurements, for children from NH (grey circles), SSD-noCI (blue triangles), SSD+CI aided (red squares) and SSD+CI unaided (yellow crosses) groups. The dotted line at 44° degrees corresponds to chance level performance; scores below the dotted line at 34° are significantly better than chance. Estimated mean scores per group (black dots) and corresponding confidence intervals (black bars) were added on top, based on the model used for the main analysis (*MAE ~ group + age + I|ID*).

All group comparisons were highly significant (p < 0.001), except for the difference between the SSD-noCI and the unaided SSD+CI group (0.2°, *p* = 1). The graph does not show the effect of age, which was significant and interacted with group.


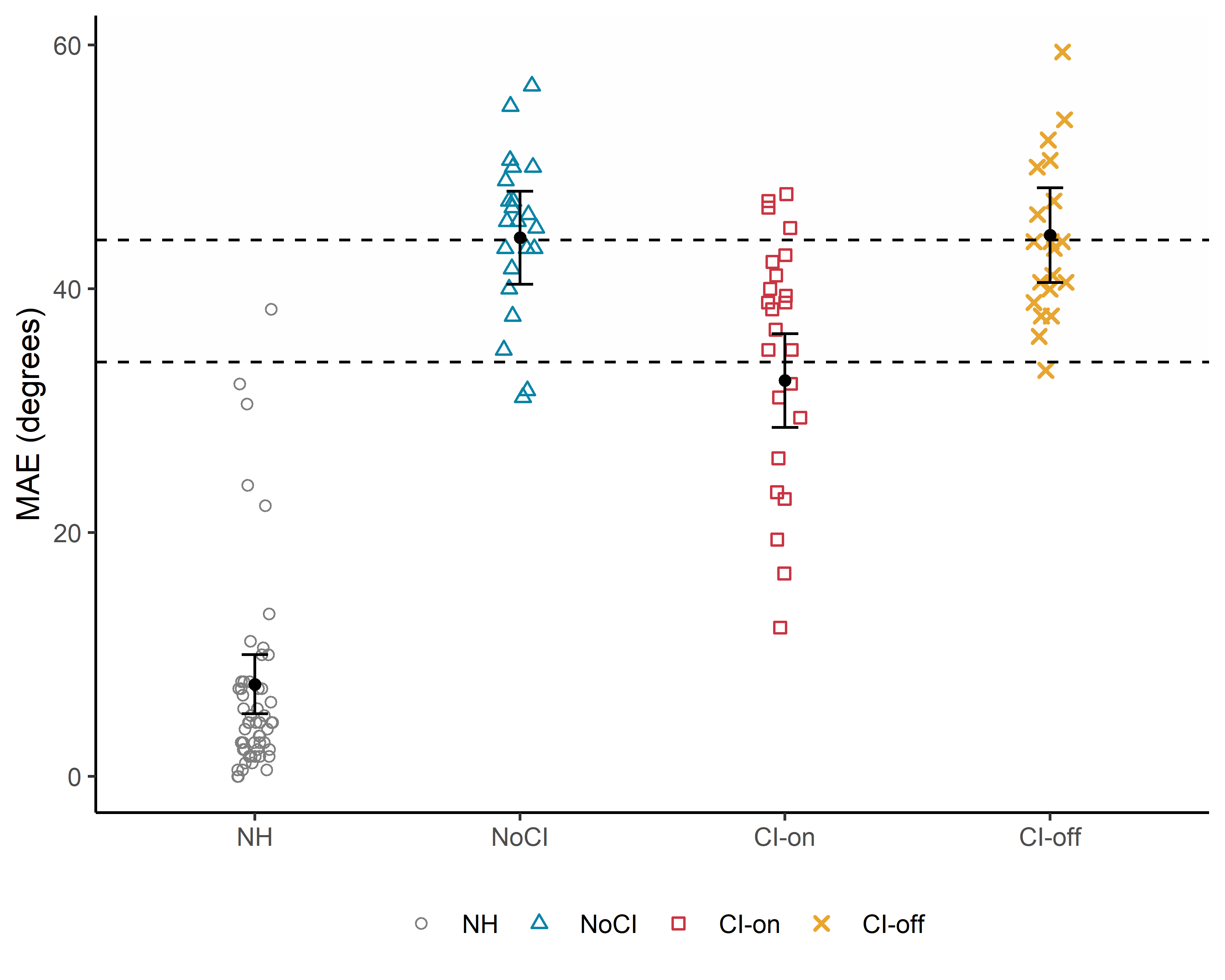

Supplement: Supplementary file 1 — Supplementary Information. [file 41598_2022_13247_MOESM1_ESM.docx]
